# Nearly three in every five prisoners experience common mental disorders (CMDs) in Jimma correctional institution; south-West Ethiopia

**DOI:** 10.1186/s12889-019-7879-6

**Published:** 2019-11-26

**Authors:** Wasihun Adraro, Habtamu Kerebih, Workinesh Tesema, Fira Abamecha, Hailemariam Hailesilassie

**Affiliations:** 1 Department of clinical nursing, Mizan Aman College of Health Science, Mizan Teferi, Ethiopia; 2 Department of psychiatry, School of Medicine, Collage of Medicine and Health Sciences, University of Gondar, Gondar, Ethiopia; 3 Department of psychiatry, College of Medical Sciences, Institute of Health, Jimma University, Jimma, Ethiopia; 4 Department of Health, Behaviour and Society, Faculty of Public Health, Institute of Health, Jimma University, Jimma, Ethiopia

**Keywords:** “Common Mental Disorders”, “Prisoners”, “Ethiopia”

## Abstract

**Background:**

Millions of people are currently suffering from some form of mental disorders. The prevalence of common disorders is twofold higher in prison than general population and the condition is found to be worst in African in general and Ethiopia in particular. The aim of this study was to assess the prevalence of common mental disorders and its correlates among prisoners in Jimma town correctional institution; Ethiopia.

**Methods:**

A cross sectional quantitative study was conducted enrolling 300 prisoners. The participants were recruited using systematic random sampling technique. The World health organization Self Reporting Questionnaire (SRQ-20) scale; was adapted to assess prevalence of mental disorders. Data were collected on interviewer administered basis by trained personnel. Statistical package for social sciences (SPSS) version 20 for widows was used for data analysis. Multivariate logistic regression modelling was conducted to identify the independent predictors of common mental disorders. *P*-value less than 5% was considered to indicate significant associations.

**Results:**

The prevalence of common mental disorder was found to be 62.70, 95% CI; [57.30, 68.50]. Prisoners who had monthly income of 1500-3000birr, AOR = 3.30, 95% CI; [1.25, 8.69] and above 3000birr; AOR = 4.75, 95% CI; [1.57, 14.33], family history of mental illness; AOR = 6.14, 95% CI; [1.65, 22.79] and who ever used Khat; AOR = 4.33, 95% CI; [2.02, 9.27] were more likely to experience common mental disorders. Conversely; having some sort of work in prison; AOR = 0.25, 95% CI; [0.12, 0.54], perceived moderate social support; AOR = 0.094, 95% CI; [0.04–0.215] and perceived moderate strong; AOR = 0.025, 95% CI; [0.009, 0.07] were found to be protective factors.

**Conclusion:**

Prevalence of common mental disorder was considerably high as nearly more than three in every five prisoner experience some form of the disorder. Early screening of prisoners up on their arrival, appropriate treatment and follow up should be done. Furthermore; creating prison based jobs that could bring prisoners together and encouraging positive relationships to build social supports as coping strategy is required.

## Background

Common mental disorders (CMDs) are group of non-communicable diseases that include anxiety, depression and somatoform disorders which are characterized by symptoms like insomnia, fatigue, irritability, forgetfulness, difficulty in concentrating and other somatic complaints [1, 2]. These disorders are found to be most prevalent in the world [3, 4] with 8.2 million and 4 million people are currently suffering from anxiety and depressive disorders respectively [5]. The prevalence among the general population seems almost the same in developed and resource poor countries with 44% [6] and 48.6% respectively [4]. The social conditions like poverty, civil war, draught and widening income inequalities are found to be major risk factors for the illness. In Ethiopia; the national prevalence of CMDs was 31.8% among general population [7].

Prisoners are the most vulnerable segment of population to various types of mental disorders. The prevalence of CMDs among prisoners was found to be twice as higher as that of the general population [8–10]. Globally; about one in nine prisoners suffer from some form of CMDs [11]. Studies conducted in South Africa 55.4%, Zambia 63.1%, Nigeria 57%, and Ghana 70% have showed high prevalence level of CMDs among prisoners [11–14].

Despite the high morbidity and disablement due to mental disorders, little attention has been received from the African governments [2, 15]. Several complex psychosocial and behavioural factors are indicated responsible for the high prevalence rate of these disorders. The prison environments like overcrowding, various forms of violence, lack of privacy, disruption, isolation from social networks, insecurity about future prospects and inadequate health services in prisons are some of prison inherent determinants of CMDs [10, 16]. Many of these disorders may be present before imprisonment or may be exacerbated by the stress related to imprisonments. Lack of social support, prison based jobs, thoughts of impossibility to run life after prison, long duration of prison life, being discriminated from families and friends and guiltiness feelings about crimes are some of exacerbating factors [8, 17, 18].

It’s been reported that imprisonment increases vulnerabilities and heightens mental ill health and require prompt care for psychological and physical illnesses Mental illness are increasing at alarming rate in the general population late alone in prison mainly due to the dynamic and changing nature of behavioral risks, economical and political conditions [19]. The nature of prison institutions-poor physical facility, inadequate space, lack of basic health services that includes counseling and treatment for management of mental disorders are supposed to affect health of prisoners. In Ethiopia; according to World prison brief report 2018, trends in prison population rates (that is, the number of prisoners per 100,000 of the national population) have increased over the last 20 years at alarming rate. It was 94, 96, 124 and 127 per 100,000 in 2001, 2005, 2011 and 2015 respectively, making Ethiopia listed top among eastern African countries [20]. These prisoners encompass individuals from different segment of population, with varying categories of crimes and sentences duration.

Addressing mental health care in prison requires comprehensive evidences for effective decision making process. It has been observed that there is paucity of evidences as only few studies were conducted in Ethiopia so far to assess prevalence of mental disorders in prisons [16, 21–24]. Moreover; except one [22]; majority of these studies were incomprehensive mainly due to the type of assessment tools employed, aspects of mental illness explored and the context of the studies [16, 23, 24]. Therefore; the aim of this current study was to assess the prevalence of common mental disorders (CMD) and its socio-demographic, and social-cultural correlates among prisoners in Jimma correctional institution; Ethiopia.

## Methods

### Study area and design

Institutional based cross-sectional study design was employed in Jimma town correctional institution from June 1, 2017 to June 30, 2017. Jimma town is located 352 km Southwest of Addis Ababa, the capital city of Ethiopia. There was one correctional institution in Jimma town currently serving for 1460 prisoners. Out of them; majority; 1418 were male prisoners and the rest 42 of them were female. The prison started functioning after the expulsion of the occupying Italian forces in 1943. The prison serves the following regions: Oromia regional state, Southern nations and nationalities, and peoples region (SNNPR) and *Gambella* region.

### Sample size and sampling technique

A sample of 302 prisoners was calculated using single population proportion formula by considering the following parameters: Proportion (P) of individuals with common mental disorders from previous study in Ethiopia, 67.60%, z = standard score corresponding to 95% confidence level to be 1.96 and d = margin of error 5% (0.05). These parameters were combine together through the equation, n = $\frac{{\boldsymbol{z}}^{\left(\boldsymbol{\alpha} /\mathbf{2}\right)\mathbf{2}}\boldsymbol{p}\ \left(\mathbf{1}-\boldsymbol{p}\right)}{d^2}$ which yields *n* = 340. This was further corrected to total prison population which was 1460 prisoners in the year 2017; yielding 302. Finally, systematic random sampling technique was employed to draw a total of 302 participants from a sampling frame consisting of 1460 prisoners.

### Data collection tools and methods

Data were collected by using a structured questionnaire through interviewer administered basis. The questionnaire had six sections. The first section was socio-demographic dimension. The other section covered assessment of mental disorders which was measured by t*he World health organization Self Reporting Questionnaire (SRQ-20) scale to assess prevalence of mental disorders* [25]. The scale addressed the overall physical, psychological and social symptoms of mental disorders among prisoners. Despite the scale was designed mostly for self-administered approaches; was also found to be suitable for interviewer administered questionnaire especially in settings with low literacy rate in developing countries like Ethiopia [26]. It has 20 items scaled as zero or one (0 or 1) each. A score of one (1) indicate that the symptom felt present during the past month and score zero (0) indicate that the symptom absent [25].

One of the challenges with this scale was its poor predictive validity across culture and context especially when the score of 7/8 cut off point is employed. This cut-off point tends to underestimate prevalence of mental disorders especially among women [27]. However; the scale demonstrated better validity in culture of developing nations including Ethiopia [26]. However; since the SRQ-20 is not a diagnostic tool; the use of cut off point doesn’t matter as it is enough to establish the mental health profile of a given population at a given time. With our study; since the aim was to screen out prisoners with suspected mental disorders, the prevalence of CMDs could be better estimated from a cut off point defined; according to the number of positive responses for symptoms. Subjects were classified as suspected cases when they had cut off point eight or more positive responses to items so that to give meaningful interpretation and explicit recommendation for policy advocacy.

Another important dimension recommended to be included in mental disorder measurements is; the social support dimension. This was measured using the Oslo-3 scale questionnaire on five point Likert scales ranging from; 1 = strongly disagree to 5 = strongly agree [28]. For the sake of convenient analysis; the score was calculated for three categories of level of social support as (< 4 = poor, 4–9 = moderate and > 9 high). The questionnaire was prepared in English and translated to a local language *Afan Oromo*. The reliability coefficient for the general OSLO-3 scale was Cronbach’s alpha of 0.73 which was considered acceptable level [29].

### Statistical data analysis

Data was checked for completeness and consistency. It was coded and entered in the computer using statistical package for social sciences (SPSS) version 20 for windows (v 20.0; IBM Corporation, Armonk, NY, USA). Descriptive statistics like frequencies, proportions, mean and standard deviations were computed. Binary and multivariable logistic regression modelling were conducted to identify determinant factors of CMDs among prisoners. Using odds ratio (OR) with 95% limit of confidence interval, the association of responseand explanatory variables was assessed and their degree of associations was computed. Finally; *p*-value less than 5% was considered to indicate significant associations.

## Results

### Background characteristics of respondents

Three hundred; out of 302 respondents have completed the study yielding 99.3% response rate. Two hundred eighty one; 281 (93.7%) participants were males. The mean age of the respondents was 30 (±12) years. Majority of the respondents; 174 (58%) were Muslims followed by Christian orthodox; 94 (31.3%). The Oromo Ethnic group encompasses majority with 199 (66.3%) followed by Amhara ethnic group; 43 (14.3%). Almost half; 157 (52.3%) of respondents were single and many; 160 (53.3%) of them had primary level educational experiences. About; 86 (26.7%) were farmers. The majority 191 (63.7%) of the respondents were living in urban before prison and 125 (41.7%) have children (Table 1).

**Table 1:** Socio-demographic characteristics of prisoners in Jimma town correctional institution, South-Western Ethiopia; June 2017 (*n* = 300)

Characteristics		Frequency	Percent (%)
Sex	Male	281	93.7
	Female	19	6.3
Age	16–25	136	45.3
	26–35	95	31.7
	36–45	44	14.7
	46–55	8	2.7
	> 56	17	5.6
Residency before prison	Rural	109	36.3
	Urban	191	63.7
Religion	Muslim	174	58.0
	Christian orthodox	94	31.3
	Protestant & catholic	32	10.7
Marital status	Single	157	52.3
	Married	126	42.0
	Others*	17	5.7
Ethnicity	Oromo	199	66.3
	Amhara	43	14.3
	Dawuro & Yem	29	9.7
	Others**	29	9.7
Educational status	Unable to read & write	41	13.7
	Grade 1–8	160	53.3
	Grade 9–12	72	24.0
	Collage and above	27	9.0
Occupational status	Farmer	80	26.7
	self employed	114	38.0
	Employed	37	12.3
	Student	36	12.0
	Others***	33	11.0
Gross monthly Income (ETB)	100–800	125	41.7
	801–1500	57	19.0
	1501–3000	69	23.0
	> 3000	49	16.3
Having children	Yes	125	41.7
	No	175	58.3

Key: *Divorced, Widowed and separately living, **Kaffa, Gurage, Tigre, Wolaita, ***Daily labour, housewife, jobless.

### Prevalence of CMDs among prisoners

**Fig. 1:**
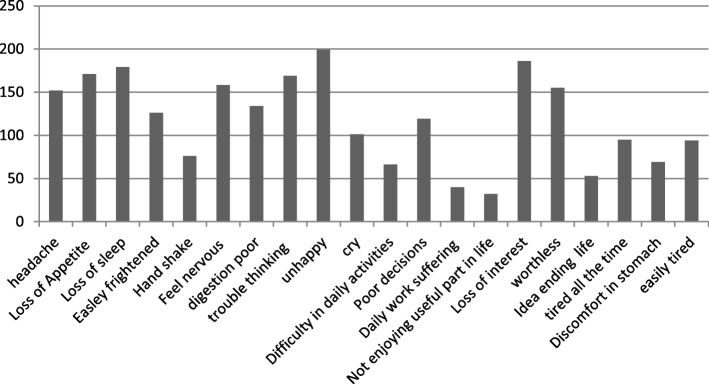
Frequency of endorsed symptoms of CMDs on SRQ-20 scale

The prevalence of CMDs among prisoners as measured on SRQ-20 scale was considerably high with 188 (62.70%); 95% CI [57.30, 68.50%]. Females were less affected as only 14 (4.70%) experienced CMDs compared to males; 174 (58%). Specifically within the SRQ-20 score; loss of interest, loss of sleep and unhappiness were indicted as major mental symptoms reported. Importantly; symptoms like trouble thinking, feeling of worthless and nervousness were also observed salient symptoms in the present study (Insert Fig. 1).

### Socio-demographic correlates of CMDs

The analysis began with bivariate logistic regression analysis in which explanatory variables with *p*-value less than 0.05 were selected for multivariate logistic regression models to control for possible confounding. Accordingly; fifteen variables namely; age category, marital status, occupation before prison, monthly income before prison, time spent in person, type of current crime committed, previous incarceration, religion, work status in prison, perceived social support, self-reported chronic physical illness, family history of mental illness, ever use of *Khat*, ever use alcohol and ever smocking were variables which demonstrated significant association with CMD in bivariate logistic regression analysis.

However on multivariate logistic regression modeling; only five variables namely; monthly income and having family history of mental illness, ever use of *Khat*, having worked in prison and social support were emerged to be significant predictors of CMDs. Accordingly; prisoners who had monthly income of 1500–3000 birr; AOR = 3.30, 95% CI; [1.25, 8.69] and above 3000birr; AOR = 4.75, 95% CI; [1.57, 14.33], family history of mental illness; AOR = 6.14, 95% CI; [1.65, 22.79] and who ever used *Khat*; AOR = 4.33, 95% CI; [2.02, 9.27] were found more likely to have common mental disorders. In-contrast; having some sort of work in prison; AOR = 0.25, 95% CI; [0.12, 0.54], perceived moderate and strong social support; AOR = 0.094, 95% CI; [0.04–0.215], AOR = 0.025, 95% CI; [0.009, 0.07] respectively; were found less likely to have common mental disorders. (Table 2).

**Table 2:** Multiple logistic regression modeling for independent predictors of CMDs among prisoners in Jimma correctional institution, South-Western Ethiopia, 2017 (*n* = 300)

Variables	Categories	CMD		AOR (95% CI)	p-value
		No	Yes		
Age category					
	16–25	56 (41.2)	80 (58.8)	Ref	Ref
	26–35	30 (31.6)	65 (68.4)	1.50 (0.87–2.63)	0.139
	36–45	16 (36.4)	28 (63.6)	1.22 (0.61–2.47)	0.571
	> 46	10 (10)	15 (60)	1.5 (0.65–3.04)	0.132
Occupation					
	farmer	32 (40)	48 (60)	Ref	Ref
	Employed	51 (33.78)	100 (66.2)	0.61 (0.32–4.21)	0.811
	Others*1	29 (42.02)	40 (57.98)	1.33 (0.54–3.24)	0.533
Monthly income	ETB				
	100–800	56 (44.8)	69 (55.2)	Ref	Ref
	801–1500	19 (33.3)	38 (66.7)	2.01 (0.74–5.47)	0.174
	1501–3000	20 (29)	49 (71)	3.30 (1.25–8.69)	0.016
	> 3000	17 (31.7)	32 (65.3)	4.75 (1.57–14.33)	0.006
Time spent in prison in months					
	< 4	25 (30.1)	58 (69.8)	Ref	Ref
	5–11	29 (34.1)	56 (65.9)	1.20 (0.63–2.30)	0.105
	12–24	32 (47.1)	36 (52.9)	0.58 (0.30–1.12)	0.416
	> 25	26 (40.6)	38 (59.4)	1.70 (0.80–6.23)	0.211
Presence of physical illness					
	No	101 (41)	147 (69)	Ref	Re
	Yes	11 (21.15)	41 (79.85)	0.93 (0.02–4.56)	0.732
Previous incarceration					
	No	107 (39.2)	167 (58.8)	Ref	Ref
	Yes	5 (19.23)	21 (80.77)	1.57 (0.33–1.41)	0.213
Type of crime					
	Robbery	25 (36.4)	44 (63.6)	Ref	Ref
	Rape	14 (48.3)	15 (51.7)	0.59 (0.18–1.96)	0.393
	Corruption	11 (61.1)	7 (38.9)	1.51 (0.64–3.54)	0.348
Work in prison					
	No	58 (26.5)	161 (73.5)	Ref	Ref
	Yes	54 (66.7)	127 (33.3)	0.25 [0.12–0.54]	< 0.001
Perceived social support					
	Poor	21 (12.5)	147 (87.5)	Ref.	
	Moderate	40 (56.3)	31 (43.7)	0.09 [0.04–0.215]	< 0.001
	Strong	51 (83.6)	10 (16.4)	0.03 [0.009–0.07]	< 0.001
Family history of mental illness					
	No	102 (42)	145 (58)	Ref	Ref
	Yes	11 (18.87)	43 (78.13)	6.14 [1.65–22.79]	0.007
Ever Khat use					
	No	92 (58.1)	69 (42.9)	Ref.	
	Yes	20 (14.4)	119 (85.6)	4.33 [2.02–9.27]	< 0.001
Ever alcohol use					
	No	98 (37.55)	163 (62.45)	Ref	Ref
	Yes	14 (35.59)	25 (64.41)	2.44 (0.96–1.34)	0.412
Smoking					
	Yes	101 (43.16)	133 (57.94)	Ref	Ref
	No	11 (14.67)	55 (83.33)	2.2 (0.71–4.18)	0.671

Key: *AOR* Adjusted odds ratio, *CI* Confidence interval, *CMD* Common mental disorders, *Ref* Reference category, *ETB* Ethiopian birr.

## Discussion

This study has used the SRQ-20 scale to assess the prevalence of CMDs and its socio-demographic correlates among prisoners in Jimma town correctional institution. The prevalence of CMDs as measured using SRQ-20 scale with cutoff point 8 and above in the 4 weeks preceding the interviews was found to be 62.70% (95% CI 57.30, 68.50). The finding was the same with reports of similar study done in Zambia showing 63.10% [11], and studies done in USA; 64% and England 65.3% [30, 31]. But the finding is considerably higher when compared to a couple of African studies; Nigeria; 57%, South Africa; 55.4% [12, 13] and a study done in Iran; 43.4% [32].

.The difference might be due to differences in assessments tool used, sample size, socioeconomic status, political context, the conditions of prisons and prison services [19]. For instance; the study conducted in Nigeria used the tool for general health questionnaire which has 28 items [13] unlike the present study which employed the SRQ-20 specific mental conditions with cut of point 8 and above. Further; the current study was done in the context of “politically motivated causes” of public unrest that sustained for more than 4 years. However; the finding of the current study was found to be lower than that of couple of studies done in Ethiopia towns; Debremarkos; 67.60% and Northwest Ethiopia; 83.40% [16, 22] and a study conducted in Ghana 70% [14]. These inconsistencies may be due to differences in sample size, assessment tool employed and socio-cultural and political contexts. For example; one of the Ethiopian study [16] has enrolled a sample of 608 participants and used Kessler Psychological Distress Scale (K10) as assessment tool.

The present study has also tried to identify the independent predictors CMDs among prisoners through multiple logistic regression modeling. It was revealed that CMDs were more likely to occur among prisoners who had a monthly income between birr 1500–3000 and above 3000 before admission to prison. The considerably higher prevalence of CMDs associated with income could be explained by disrupted means of in income during their stay at prison. This finding was comparable with that of study done in South Africa [15] and it was different from study conducted in Nigerian prisoners [13]. The reason might be related to prisoners handling, control prisons and availability of psychoactive substances as those who have good income had an ability to buy and use psychoactive substance in illegal way during their stay at prison and to develop mental illness [13].

It was evidenced lack of work is aggravating factor for stress which in turn become risk factor for mental illness [28, 33]. In the current study having work in prison was found to be a protective factor for CMDs as about 75% of respondents who had work in prison were less likely affected by CMDs. It was explained that loss of work itself has biological, social and psychological influence on individuals [34, 35]. In the same way; having work has its own effect on decreasing depression, anxiety and related physical complaints [36]. Further; more work is considered one source of income and social supports and these two variables were identified important predictors of CMDs in this study.

The social support is an important variable that plays a beneficial role in the maintenance of mental health and psychological wellbeing. To this regard, social support is thought to relive the effects of stress by enhancing personal coping abilities such as self-esteem and self-efficacy. Through a strengthening of the coping mechanism, the negative emotional reaction to a stressful event will either be reduced, or the physiological responses on health via the immune system will be dampened [28, 34]. In the current study respondents who had or perceived having moderate and strong social support were less likely affected by CMDs which was consistent with findings from similar studies of different countries like Iran; Egypt, and Norway and [28, 33, 37].

The common use of psychoactive substances before imprisonment is a serious public health problem because of its consequences of committing crime, behavioral disturbances and the persistence guilty feeling towards imprisonment [38]. Various types of substances that include *Khat,* alcohol and cigarettes are very much common in our countries causing significant health and behavioral problems among users [6, 34]. In the present study; CMDs was more than four times higher in prisoners who had history of ever using *Khat* which was in line with previous study conducted among homicidal offenders in Jimma town prison [23] and study done among residents of Jimma town [39]. The similarity might be due to the fact that Jimma is the second most *khat* growing area in Ethiopia next to Hararghe (a region located eastern part of Ethiopia) and hence easily accessible for everybody [39, 40].

Consistently with the previous study [40]; family history of mental illness was found to be best predictor of CMDs in which prisoners whose family had mental illness were six times more likely to have CMDs. It has been revealed that mental illness in family goes to the next generation and the process of illness was also the risk factor for committing crime and admission to prison [34]. The priori presence of some form of CMDs may be further exacerbated by the stress caused of imprisonment. The exacerbating factors are many; lack of social support, lack of income generating job, frequent thought of impossibility to run life after prison, long duration of prison life, guilt feelings about incarceration and not enjoying meaningful life are few among many [8, 17, 18].

### Limitation of the study

First; the study has employed cross-sectional study design in which it’s difficult to establish cause and effects relationships. Due to the vulnerable nature of the study setting; the possible influence of social desirability bias on the result should be considered while interpreting the finding. On top of this; the study couldn’t examined the pre-existing mental illness and hence should be considered as limitation. Furthermore; majority of scales previously used for the assessment of mental health status at clinical and community settings performing varying degree of validity across settings, populations and cultures. These scales are the Beck Depression Inventory (BDI-II) scale, the structured clinical interview for depression in DSM-5, comprehensive psychopathological rating scale, and the SRQ-20 were few among many [27, 41–43]. Despite it has many limitations; the SRQ-20 demonstrated better validity in culture of developing nations including Ethiopia [26]. These limitations are primarily related to the type of analysis method employed. The cut off point method (Score > 8) which was considered in the current as well; does not provide detailed knowledge of the contribution of each dimension of depression/anxiety, somatic symptoms, reduced vital energy, depressive thoughts. However; since the SRQ-20 is not a diagnostic tool; the use of cut off point doesn’t matter as it is enough to establish the mental health profile of a given population at a given time. Furthermore; study have showed that the cut off point SRQ-20 tends to underestimate prevalence of mental disorders among women [27].

## Conclusion

Prevalence of CMDs in the prison was considerably high as nearly over three in every five prisoner experience some form of CMDs. Behavioural factors like using of *khat* in life time and family history of mental illness were emerged to be risk factors for CMDs while perceived social support, varying degree of income and engaging in some sort of work in the prison were identified preventive factors to CMDs.

### Recommendations

Recognitions and assessment of prisoners for CMDs up on their arrival and targeted follow up screening and treatment should be done regularly. Further; creating some sort of jobs that could bring prisoners together and encouraging them to create positive relationships so as to build sense of social supports and self-efficacy to coping with possible stresses is required. Furthermore, large scale longitudinal research should be launched to track the process of CMDs development and infer the causal and effects.

## Data Availability

The datasets used and analyzed during the current study is available from the corresponding author on reasonable le request.

## Abbreviations

AOR: Adjusted odds ratio
CMD: Common mental disorders
K10: Kessler Psychological Distress Scale
SNNPR: Southern nations and nationalities, and peoples region
SPSS: Statistical Package for Social Science
SRQ: Self reporting questionnaire
SRQ-20: Self-response questionnaire consisting 20 items
WHO: World Health Organization
